# Forecasting Energy Market Contracts by Ambit Processes: Empirical Study and Numerical Results

**DOI:** 10.1155/2014/879892

**Published:** 2014-10-28

**Authors:** Luca Di Persio, Michele Marchesan

**Affiliations:** Department of Computer Science, University of Verona, Strada le Grazie 15, 37134 Verona, Italy

## Abstract

In the present paper we exploit the theory of ambit processes to develop a model which is able to effectively forecast prices of forward contracts written on the Italian energy market. Both short-term and medium-term scenarios are considered and proper calibration procedures as well as related numerical results are provided showing a high grade of accuracy in the obtained approximations when compared with empirical time series of interest.

## 1. Introduction

In recent years the economy of energy markets has been interested by deep transformations due to political as well as technological changes all over the world. Such innovations have been often characterized by procedures of liberalisation which have often led to the creation of completely new markets as in the case of, for example, the Nordic Nord Pool market, the German EEX market, the Italian GME, and so forth, most of which are animated by a plethora of different financial products such as spot or forward/futures contracts, European options, and exotic options.

Although energy markets seem similar to classical financial ones, there are many differences between them. The electricity spot cannot be stored directly, or, at least, only a small quantity can be kept using reservoirs for hydrogenerated power or exploiting large and expensive batteries. This makes the supply of power very inelastic as it is influenced by seasonal, weekly, and intradaily pattern, resulting in a very illiquid framework.

Latter characteristics give rise to incompleteness of the energy markets; hence classical mathematical approaches, such as those which are based on the Brownian setting, for example, the Black and Scholes model (see [1]) are not satisfactory; see, for example, [2, 3] and references therein.

To overcome (at least some of) such drawbacks, the theory of ambit processes has been proposed by Barndorff-Nielsen and Schmiegel; see [4], where the authors studied some type of turbulence's problems which was later applied to analyse energy markets and then improved to take into account related financial products; see, for example, [5–7]. Let us underline the fact that ambit processes provide a flexible class of random field models where we can easily incorporate leptokurtic behaviors in returns, stochastic volatility, seasonal pattern, and the observed Samuelson effect; see, for example, [8] and references therein, for a treatment of such an economical effect.

Ambit processes (see Section 2 and references therein for details) are characterized by a Lévy basis and a deterministic function, integrated on an interval called* ambit set*. They allow specifying directly the model based on a probabilistic understanding of the phenomena; moreover they are well defined under weak integrability conditions. We would also like to underline the fact that the theory of stochastic differential equations driven by Lévy processes has been widely used in finance providing an effective set of techniques used to model a huge quantity of different financial scenarios; see, for example, [9–12] and references therein.

In this work we briefly present the theory of ambit processes emphasizing how they can be applied to the study of energy markets' forward prices. In Section 2 we show the concepts of ambit process and Lévy basis, focusing our attention on those results which will be relevant to model, see Section 3, the forward price problem; see also, for example, [13–15] for related financial setting; then, in Section 4, we concretely apply the proposed approach to forecast the price of a particular forward contract written within the Italian energy market; calibration of relevant parameters as well as short-term and medium-term analysis is also provided.

## 2. Ambit Processes

This section is intended to be an introduction to stochastic ambit processes; for a deeper treatment of the subject we refer to [5–7] and references therein.

A rigorous treatment of ambit fields and ambit processes is based on the definition of* Lévy basis*. A homogeneous Lévy basis is a special class of independently scattered random measures (ISRM); see, for example, [16]. We denote by (*Ω*, *ℱ*, *P*) a general probability space; let *S* ∈ *ℬ*(*ℝ* ^*k*^) be a subset of the class of Borel sets of *ℝ* ^*k*^, *k* ∈ *ℕ* ^+^, and let us denote by *ℬ*_*b*_(*S*) the class of bounded Borel subsets of *S*. The following definitions will be used later on; for more details we refer to [17].

**Definition 1 .**

A family {Λ(*A*) : *A* ∈ *ℬ*_*b*_(*S*)} of random vectors in *ℝ* ^*d*^ is called *ℝ* ^*d*^-valued Lévy basis on *S* if the following three properties are satisfied.

(1) The law of Λ(*A*) is infinitely divisible for all *A* ∈ *ℬ*_*b*_(*S*).
(2) If *A*_1_,…, *A*_*n*_ are disjoint subsets in *ℬ*_*b*_(*S*), then Λ(*A*_1_),…, Λ(*A*_*n*_) are independent.
(3) If *A*_1_, *A*_2_,… are disjoint subsets in *ℬ*_*b*_(*S*) with ⋃_*i*=1_^*∞*^ *A*_*i*_ ∈ *ℬ*_*b*_(*S*), then
  (1) $$\begin{array}{c} \Lambda \left(\underset{n\to \infty }{\lim}\overset{n}{\underset{i=1}{⋃}}A_{i}\right)=\overset{\infty }{\underset{i=1}{\sum }}\Lambda \left(A_{i}\right),\;\;\text{a}.\text{s}., \end{array}$$
 with respect to ISRM.

**Definition 2 .**

An ambit process is defined as a solution, *Y*_*t*_(*x*), of the following stochastic equation called ambit field:

(2) Yt(x)=μ+∫At(x)g(ξ,s;x,t)σs(ξ)L(dξ,ds)+∫Dt(x)q(ξ,s;x,t)as(ξ)dξds,

where *A*_*t*_(*x*), *D*_*t*_(*x*) are ambit sets; *g* and *q* are deterministic functions of space and time; *σ* is a stochastic and positive random field, often referred to as volatility; and *L* is a Lévy basis.

In order to define the proper filtration to work with, let *L* be a Lévy basis on *S* × [0, *T*] ∈ *ℬ*(*ℝ* ^*k*+1^), for some *k* ∈ *ℕ* ^+^ and a given finite time horizon *T* ∈ *ℝ* ^+^; then for any *A* ∈ *ℬ*_*b*_(*S*) and *t* ∈ [0, *T*], we define (see [5]) the following measure-valued processes: *L*_*t*_(*A*): = *L*(*A*, *t*) = *L*(*A* × (0, *t*]), which can be used as integrator (see [18]) under the square-integrability assumption. Exploiting the latter definition we can define the filtration {*ℱ*_*t*_}_*t*∈[0,*T*]_, as follows:

(3) Ft=∩n=1+∞Ft+1/n0,with  Ft0=σ{Ls(A):A∈Bb(S), 0<s≤t}∨N,

where *𝒩* denotes the *ℙ*-null sets in *ℱ*; see Section 3.3 of [5].

In many applications we are interested in, it is useful to consider ambit processes that are stationary in time and nonanticipative. The ambit set *A*_*t*_(*x*) is homogeneous and* nonanticipative* if it is taken of the form *A*_*t*_(*x*) = *A* + (*x*, *t*), where *A* only involves negative time coordinates. If *g*, *σ*, *q*, and *a* are sufficiently smooth to allow for the existence of the integrals in (2), then they can be defined in the sense of ISRM; see, for example, [19], for a detailed treatment of Lévy integration.

### 2.1. Modelling Forward Price Using Ambit Processes

Due to their structure, ambit fields can be used to capture many of the peculiarities characterizing modern energy markets, for example, their strong seasonal patterns, very pronounced volatility clusters, high spikes/jumps, the so-called Samuelson effect, and so forth. We recall that the Samuelson effect describes the fact that the volatilities of the forward price are generally smaller than the ones of the underlying spot price; moreover such volatilities quickly converge to the volatility of the spot itself, when time to maturity tends to zero.

The forward price case requires the introduction of a component which could be able to reflect the fact that the forward price depends also on the* time to maturity*. Such a component is modelled as an ambit field, with both temporal and spatial component, of the following form:

(4) $$\begin{array}{c} f_{t}\left(x\right)=\int_{A_{t}(x)}k\left(\xi ,t-s;x\right)\sigma_{s}\left(\xi \right)L\left(d\xi ,ds\right), \end{array}$$

where *t* ≥ 0 denotes the current time, *T* > 0 denotes the maturity time, and *x* = *T* − *t* indicates the corresponding time to maturity. Note that in (4), *σ*_*s*_(*ξ*) > 0 is a stochastic field on *ℝ*_+_ × *ℝ*, which is stationary in the time domain and such that it expresses the volatility on the forward market as a whole.

According to Definitions 1 and 2 and (2) a specific model can be built specifying the sets *A*_*t*_(*x*), the damping function *k*, and the stochastic volatility field *σ*_*s*_(*ξ*). In each case the choice will be based on market intuition and consideration of mathematical tractability; for example, in (4), it is convenient to assume that *σ* is independent of *L*. Moreover in order to ensure that *f*_*t*_(*x*) is stationary in time *t*, the ambit sets are taken to be of the form *A*_*t*_(*x*) = *A*_0_(*x*)+(0, *t*).

Let us describe a possible model for electricity forward price exploiting equation (4). In what follows we will denote by *P* ^*^ a risk neutral probability measure, which is not assumed to be uniquely determined. We shall work directly under the *P* ^*^ measure in order to ignore any drift terms, hence working with the zero-mean specification of the ambit field.

In formula (4) the forward price is characterized by:

(i) a square integrable Lévy basis *L* with mean equal to 0;
(ii) a stochastic volatility field *σ* assumed to be adapted to the filtration {*ℱ*_*t*_}_*t*∈[0,*T*]_ previously defined and independent of the Lévy basis *L*; in order to ensure stationarity in time, we assume that *σ*_*s*_(*ξ*) is stationary in *s*;
(iii) a nonnegative kernel function *k* such that it satisfies *k*(*ξ*, *u*, *x*) = 0 for *u* < 0;
(iv) a convolution *k*∗*σ* that is integrable with respect to *L*;
(v) a chosen ambit set *A*_*t*_(*x*) of the type *A*_*t*_(*x*) = *A*_0_(*x*)+(0, *t*).

It turns out that, to construct the model, we have to specify the kernel function *k*, the stochastic volatility *σ*_*s*_(*ξ*), and *L*. For a detailed analysis of the stochastic volatility part we refer to [7], while, in the next subsections, we will take into account the specification of both a particular Lévy basis and a specific kernel function.

#### 2.1.1. Specification of the Lévy Basis

Our model is based on a Lévy basis which is square integrable and has zero-mean; hence we can choose any infinitely divisible distribution satisfying these two assumptions. A very natural choice is represented by the Gaussian Lévy basis which results in a smooth random field; alternatively, we can choose a normal inverse Gaussian (NIG) Lévy basis or a tempered stable Lévy basis; see, for example, [5].

Let us recall that if the zero-mean assumption is relaxed, then we can use a gamma or inverse Gaussian Lévy basis, so that the positivity of forward price is guaranteed.

#### 2.1.2. Specification of the Kernel Function

The kernel function *k* plays a key role in our model; indeed it

1. completely determines the tempospatial autocorrelation structure of a zero-mean ambit field,
2. characterises the Samuelson effect,
3. determines whether the forward price is a martingale.

Recall that *k* is a function of the variables *ξ*, *t* − *s*, *x*, where *t* − *s* is the temporal component, while *ξ*, *x* are the spatial ones.

A rather natural approach to specify a kernel function is to assume a factorisation property. We will present two different types, which are important in different contexts. In the first example the kernel is subdivided into a spatial and a temporal component. The kernel factorises as follows:

(5) $$\begin{array}{c} k\left(\xi ,t-s,x\right)=\phi \left(\xi ,x\right)\psi \left(t-s\right), \end{array}$$

for suitable functions *ψ* and *ϕ* representing the temporal and the spatial component respectively. For the temporal kernel part, we can choose an exponential function, a choice which is motivated by an Ornstein-Uhlenbeck process approach; otherwise a more generally option is given exploiting a* continuous-time autoregressive moving average* (CARMA) process. For the spatial component, which determines the correlation between various forward contracts, we can choose a function similar to the one adopted for the temporal component.

An alternative factorisation of the kernel function is given by

(6) $$\begin{array}{c} k\left(\xi ,t-s,x\right)=\Phi \left(\xi \right)\Psi \left(t-s,x\right). \end{array}$$

Despite the fact that a factorisation does not look very natural, it is still very useful to formulate martingale conditions for the forward price since it naturally includes cases when *t* does not play an explicit role in the sense that $\Psi (t-s,x)=\tilde{\Psi }(t-s+x)=\tilde{\Psi }(T-s)$ for a suitable function $\tilde{\Psi }$.

We would like to underline the fact that the ambit processes characteristics are also very useful to study the dependence structure between various forward contracts. In particular, the autocorrelation structure is determined by three factors: the intersection of the corresponding ambit sets, the kernel function, and the autocorrelation structure of the stochastic volatility field. Thanks to the flexibility of ambit field we can construct different type of autocorrelation. Moreover it is possible to model different types of commodities forward or futures contracts, such as electricity and natural gas futures, simultaneously; see [5].

## 3. The Model

In this section we exploit the theory described in the previous section to propose a model, inspired by the analysis developed in [5], to simulate the forward price. As mentioned before the specifications of parameters for an applied ambit processes based model will be essentially done by interplay between mathematics aspects, numerical tractability, and empirical evidence. We start considering the following stochastic equation for the forward price process *f* = *f*_*t*_:

(7) $$\begin{array}{c} f_{t}\left(x\right)=\overset{x_{2}}{\underset{x_{1}}{\int }}\phi \left(\xi ,x\right)\psi \left(t,T\right)L\left(d\xi ,dx\right), \end{array}$$

where the kernel function is factorised following the one proposed in formula (5), the integration interval depends on how long the contract is traded in the market, and *T* > 0 is the maturity time.

In the model proposed with (7) the stochastic volatility is not used. The price of forward contracts that were studied shows a very regular trend; the introduction of stochastic volatility increases the complexity of the model but it is not required by empirical evidences. For this reason we have followed what is suggested in [5] and we have not introduced it.

In order to choose the* right* kernel function for the model in (7) we first consider the time to maturity component. As a general fact, forward contracts tend to depreciate getting close to maturity time, but the behavior of prices may be very different. In particular forward contracts are characterized by patterns that cannot be reduced to a generic convex-concave description. In our analysis, we have chosen a linear fall in price to mediate different behaviors. So, for time to maturity part of the kernel we have

(8) $$\begin{array}{c} \psi \left(t,T\right)=f_{0}-C\frac{t}{T}, \end{array}$$

where *f*_0_ is the price of forward at time 0 and *C* ∈ *ℝ* is a constant computed as the average fall in price at maturity time of a forward and, in our example, we calculated the mean value over contracts of last year; see Section 4.

We would like to underline the fact that estimates obtained using (8) are in general more accurate than those computed by a negative exponential approach to depreciation. The latter fact is particularly evident for contracts that present a sort of concave trend; see Table 6.

Now we focus on the other component of kernel function. Since electricity's price is strongly affected by seasonality, then also the forward price depends on the particular period of the year we want to consider. The latter fact guides us to choose the component *ϕ* in order to take into account the seasonal influences that affect energy markets:

(9) $$\begin{array}{c} \phi \left(\xi ,x\right)=-\frac{\sin\left(x_{1}\left(\xi \right)\right)}{K}+\frac{\left(K+\sin\left(x\right)\right)}{K}. \end{array}$$

Let us note that in (9) there are two important parameters: *x*_1_, which depends on the* space component ξ* through proper calibration procedure as pointed out in Section 4.1, and *K*. The latter constant is very interesting because it depends only on the length of the forecast. In particular short-term forecasts are less influenced by seasonality than medium or long time previsions. It follows that the parameter *K* plays the role of a* trigger* that can be used to increase or to reduce the relevance of such an effect, hence taking value according to our particular forecast interests. We also note that the definitions of the functions *ϕ* and *ψ* ensure that the forward price is a local martingale under *ℙ* ^*^; see [5, Section 6.1].

Concerning the task of choosing the most suitable Lévy basis, we note that forward price shows a certain continuity in the trend; hence we decided to use a continuous one. Due to its small daily variability we choose a distribution with small variance. In order to use the* right distribution*, we note that forward prices show a small variability; hence there are no* fat tail* phenomena to be taken into account, at least at such a time scale, we need a symmetric distribution in order to better fit related time series and we suppose that the (daily) small variations in the forward price component depend on a certain number of hidden variables whose global effect can be efficiently summarized by a* central limit theorem* approach. Therefore we set

(10) $$\begin{array}{c} L\sim N\left(\mu ,\Sigma \right), \end{array}$$

where

(11) μ=[11]  Σ=[σxdx00σtdt],

where *σ*_*x*_ and *σ*_*t*_ are constants which measure the variability on time and on time to maturity.

## 4. Empirical Study

In this part we report the concrete implementation of our model which will be tested on the behaviour of a particular type of forward traded in* Italian over-the-counter* (OTC) market, namely, a* monthly peak forward* contract which assures the supply of electricity at a fixed price for a month from 9 a.m. to 8 p.m. and only during working days. We analyse 12 forwards, one for each month of the year, starting from the first for April 2012 until March 2013. In what follows we describe how we have calibrated the model, and we present the results obtained for a short- and a medium-term forecast of the trend of these contingent claims.

### 4.1. Calibration

In this section we show how the parameters characterizing (7) have been computed, with particular emphasis on how to estimate the length of the integration.

Let us underline the fact that, with respect to (9), we have used a periodic function to simulate seasonality; namely, we have decided to use the following values for *x*_1_ = *x*_1_(*ξ*) depending on the component *ξ* which spans the different months of the year.

Let us note that values in Table 1 has to be considered taking into account that the starting point 0 is in April instead of January because each contract begins to be traded 3 months before its maturity time; therefore forward for April 2012 is traded from January 1, 2012, to March 31, 2012.

Parameter *K*, in formula (9), has been theoretically discussed before; see Section 2.1.2; here we suggest only the values we chose in our approximation. For a 5-day prediction (short-term prediction) we put *K* = 200; to predict the behavior of forward during the period when it is traded (3 months) we set *K* = 30. As we have already underlined, the parameter *K* is used to model seasonality. The importance of seasonality increases enlarging the estimated period; hence for short-term analysis we set a bigger value of *K* in order to reduce the effect of seasonality on the simulated price. The latter means that, from a concrete point of view, the chosen values for *K* have to be tailored on the specific market; hence there is not a theoretical approach able to model *K* for general frameworks.

Note that parameter *C* in formula (8) is estimated as the mean values of differences between the first and the last quotation for all monthly forward in 2012, which leads to *C* = 4. Let us note that a* least squares approach* to the estimation of *C* has shown a mean equal to 3,97, with a standard deviation of 3,12; nevertheless such values are not particularly meaningful because of the smallness of data sample. The latter fact has suggested to try for different values of *C* in a reasonable small neighbourhood of its estimated mean. Following such an approach we compared results obtained for different values of *C*, without appreciating substantial differences in short-term analysis (see Table 4 and (6)), but, see Table 6, for medium-term analysis the best forecast results have been produced taking *C* = 4. The constants *σ*_*x*_ and *σ*_*t*_ measure the daily variability over time and over time to maturity. Forward contracts show very small daily variations and empirically evidences suggest taking *σ*_*x*_ = 0.005 and *σ*_*t*_ = 0.000001.

### 4.2. Short-Term Analysis

As mentioned before, the short-term forecasts are very little effected by seasonality; hence for such type of analysis we have to decrease seasonal effect in our model. Here we present results obtained for 1 to 5 days ahead prediction. In particular Table 2 reports, as the mean obtained over 5 tests, the average absolute percentage error with respect to the number of days, from 1 to 5, for which the different forecasts are computed.

Concerning latter analysis, it is interesting to note that even if the error tends to grow in the number of days as we can expect, it remains small. The prevision on the 1st day ahead is always less than 1% and forecasts on 5 days ahead only once overtake 1,5%. Let us note that such stability is due not only to the precision of the method we proposed, but also it depends on the relative stationarity of forward contracts' trend; nevertheless it is important to underline the fact that it can be appreciated exploiting the small variance of the chosen Lévy basis.

Coherently with the qualitative analysis for the parameter *C* that we have given at the end of the previous section, we report, see Tables 3 and 4, some computations concerning different values of *C*.

**Figure 1:**
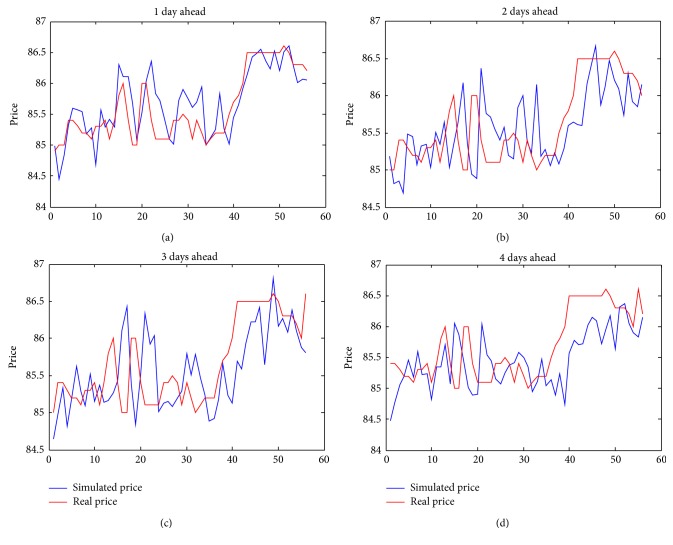
Forward September.

**Figure 2:**
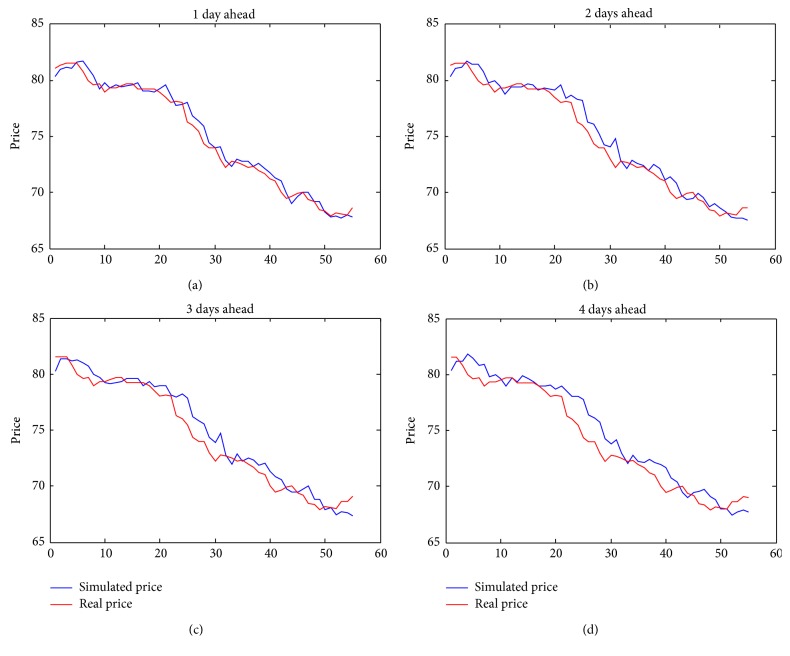
Forward March.

Figures 1 and 2 show the month with the best predictions (September) and the one with the worst ones (March). We present also previsions for 1, 2, 3, and 4 days ahead for each month.

Concerning the graphs in Figures 1 and 2, we would like to underline the fact that they are made with respect to different scales on the price component; hence they may appear to be in contrast with results obtained in Table 2, but this is not. Let us also note that small oscillations of forward for September give us better forecasts. We would like to stress that our model is able to follow rather closely the trend of real price in both September and March forwards.

### 4.3. Medium-Term Analysis

In this part we present results obtained with a medium-term analysis.

In short-term analysis we took the price today to forecast the value of the contract some days ahead. In what follows we use the first quotation of each forward to predict the whole trend. In this case a very important role is played by seasonality. In particular for a 3-month period we cannot ignore seasonal effect, as we have done in our short-term analysis. In Table 5 we present the average absolute percentage error obtained for each contract.

As we can easily guess, errors increase enlarging the period to forecast but our numerical results show that these errors can be controlled resulting in discrepancies which, in some cases, remain near the ones obtained in the short-term analysis. Note that, contracts with bigger error are often the same of those in Table 2, with the only exception for July 2012, where we have a smaller error than in the previous example. In Table 6, we compare the results obtained varying the parameter *C* and we also show (see the specification of the model at the beginning of Section 3) the estimates that we can obtain substituting the function *ψ*, defined in (9), with a negative exponential function.

**Figure 3:**
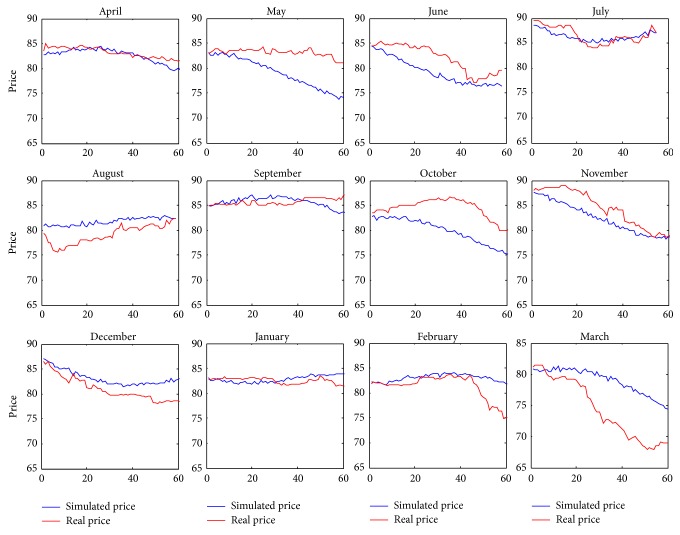
Different simulated forward.

In Figure 3 we compare the behavior of real price and the simulated one for every forward contract in order to point out the accuracy of our model which is able to closely follow the real trend for each month of the year.

In particular, in Figure 3, we can note that forwards with a bigger error are the ones which are more complicated to forecast; moreover we note that the worst previsions are not necessary at the end of the period, as we may expect.

Table 7 presents the error divided into 3 periods.

We can note that, generally speaking, better previsions are the ones made for the first period; then they tend to get worse, but this does not always happen. In particular, see, for example, what happened in July 2012; our model is able to behave correctly, namely, obtaining opposite results, and this is due to the fact that the proposed procedure is good for approximating seasonality effects.

## 5. Conclusions

In this work we have presented a model based on ambit processes to forecast forward prices both in short- and in medium-term scenarios.

In the short-term framework we have calibrated the model reducing the effect of seasonality. Errors associated with our estimates are very small; moreover we are able to control their growth when the number of days ahead increases. Our analysis shows that, comparing discrepancies with empirical data and with the related errors for the spot price, provided forecasts for forward prices are closer to reality than the ones obtained in the spot case. The latter fact is mainly due to the greater stability of the behavior of prices in the forward case compared to the spot setting, a characteristic that is particularly pointed out exploiting ambit processes.

In medium-term analysis we have tried to forecast the whole trend of a forward contract using only its first value. As we expect, errors increase if compared with the ones obtained in the short-term analysis, but some analogies have to be underlined; for example, contracts with bigger error in medium-term analysis are the same as in the short-term case. In particular our approach is able to handle abrupt changes in the real trend of electricity price with good control on estimating errors. In the medium-term study we have also included the seasonality effect, obtaining estimates that follow the pattern of forward price during all the analysed periods. Sometimes it also happens that predicted values in last part of the interval are better than initial ones, hence confirming, also exploiting related graphical analysis, that our model can give a good approximation of the seasonal pattern of the price.

## Figures and Tables

**Table 1:** Integration intervals.

Month	*x*_1_
January	11/8*π*
February	7/4*π*
March	1/8*π*
April	0
May	3/8*π*
June	3/4*π*
July	9/8*π*
August	3/2*π*
September	15/8*π*
October	1/4*π*
November	5/8*π*
December	*π*

**Table 2:** Short-term results.

Month	1	2	3	4	5
April 2012	0,47%	0,58%	0,51%	0,52%	0,54%
May 2012	0,52%	0,66%	0,75%	0,79%	0,73%
June 2012	0,67%	0,75%	0,77%	1,01%	1,15%
July 2012	0,51%	0,61%	0,80%	0,92%	1,19%
August 2012	0,63%	0,85%	0,98%	1,10%	1,17%
September 2012	0,52%	0,50%	0,46%	0,51%	0,45%
October 2012	0,28%	0,38%	0,55%	0,75%	0,85%
November 2012	0,64%	0,67%	0,75%	0,86%	0,96%
December 2012	0,51%	0,69%	0,71%	0,78%	0,90%
January 2013	0,41%	0,45%	0,55%	0,59%	0,58%
February 2013	0,70%	0,84%	1,04%	1,23%	1,41%
March 2013	0,61%	0,91%	1,21%	1,39%	1,74%

**Table 3:** Short-term results with *C* = 3.

Month	1	2	3	4	5
April 2012	0,49%	0,56%	0,54%	0,58%	0,63%
May 2012	0,5%	0,66%	0,76%	0,74%	0,65%
June 2012	0,66%	0,8%	0,86%	0,91%	1,09%
July 2012	0,45%	0,6%	0,76%	1,06%	1,21%
August 2012	0,69%	0,91%	1,01%	1,08%	1,18%
September 2012	0,44%	0,53%	0,49%	0,48%	0,49%
October 2012	0,36%	0,45%	0,61%	0,73%	0,83%
November 2012	0,63%	0,69%	0,75%	0,88%	0,92%
December 2012	0,52%	0,67%	0,82%	0,83%	0,86%
January 2013	0,44%	0,51%	0,55%	0,61%	0,7%
February 2013	0,65%	0,8%	1,08%	1,22%	1,42%
March 2013	0,65%	0,87%	1,26%	1,48%	1,8%

**Table 4:** Short-term results with *C* = 5.

Month	1	2	3	4	5
April 2012	0,47%	0,56%	0,52%	0,6%	0,56%
May 2012	0,55%	0,7%	0,78%	0,79%	0,83%
June 2012	0,66%	0,86%	0,86%	1,02%	1,08%
July 2012	0,44%	0,64%	0,73%	1,04%	1,24%
August 2012	0,71%	0,86%	0,95%	1,09%	1,27%
September 2012	0,47%	0,5%	0,43%	0,48%	0,47%
October 2012	0,35%	0,41%	0,58%	0,79%	0,89%
November 2012	0,64%	0,77%	0,73%	0,94%	0,97%
December 2012	0,61%	0,63%	0,76%	0,79%	0,89%
January 2013	0,44%	0,47%	0,53%	0,63%	0,6%
February 2013	0,75%	0,83%	0,99%	1,18%	1,28%
March 2013	0,64%	0,87%	1,15%	1,36%	1,59%

**Table 5:** Medium-term results.

Month	AAE%
April 2012	0,90%
May 2012	4,80%
June 2012	3,33%
July 2012	1,11%
August 2012	3,31%
September 2012	1,37%
October 2012	5,29%
November 2012	2,37%
December 2012	2,83%
January 2013	1,17%
February 2013	2,27%
March 2013	6,65%

**Table 6:** Medium-term analysis with different methods.

Method	*C* = 3	*C* = 4	Neg. Exp.
April 2012	0,83%	1,32%	0,91%
May 2012	4,23%	5,34%	4,85%
June 2012	2,77%	3,92%	3,72%
July 2012	1,27%	1,04%	1,38%
August 2012	3,99%	2,74%	3,36%
September 2012	1,25%	1,48%	1,47%
October 2012	4,7%	5,96%	5,42%
November 2012	2,03%	2,98%	2,76%
December 2012	3,35%	2,32%	2,71%
January 2013	1,58%	0,96%	1,30%
February 2013	2,9%	1,78%	2,72%
March 2013	7,28%	5,95%	6,47%

**Table 7:** Medium-term analysis.

Month	1st month	2nd month	3rd month
April 2012	1,05%	0,49%	1,36%
May 2012	1,34%	4,76%	8,50%
June 2012	2,75%	5,15%	2,16%
July 2012	1,51%	0,88%	0,69%
August 2012	4,90%	3,22%	1,83%
September 2012	0,63%	1,38%	1,91%
October 2012	2,40%	6,42%	7,07%
November 2012	2,71%	3,52%	1,15%
December 2012	1,43%	2,40%	4,51%
January 2013	0,83%	1,01%	1,66%
February 2013	0,86%	0,66%	5,01%
March 2013	1,46%	7,48%	10,71%
